# Binding deficits in visual short‐term memory in patients with temporal lobe lobectomy

**DOI:** 10.1002/hipo.22998

**Published:** 2018-11-19

**Authors:** Nahid Zokaei, Matthew M. Nour, Annie Sillence, Daniel Drew, Jane Adcock, Richard Stacey, Natalie Voets, Arjune Sen, Masud Husain

**Affiliations:** ^1^ Department of Psychiatry, Oxford Centre for Human Brain Activity, Wellcome Centre for Integrative Neuroimaging University of Oxford Oxford UK; ^2^ Department of Experimental Psychology University of Oxford Oxford UK; ^3^ Institute of Psychiatry, Psychology, and Neuroscience Kings College London London UK; ^4^ Nuffield Department of Clinical Neurosciences, Oxford NIHR Biomedical Research Centre University of Oxford Oxford UK; ^5^ Oxford University Hospitals NHS Foundation Trust, University of Oxford Oxford UK; ^6^ Oxford Epilepsy Research Group Oxford University Hospitals, NHS Foundation Trust, University of Oxford Oxford UK

**Keywords:** Binding, Medial Temporal Lobe, Short term memory

## Abstract

Classical views of the medial temporal lobe (MTL) have established that it plays a crucial role in long‐term memory (LTM). Here we demonstrate, in a sample of patients who have undergone anterior temporal lobectomy for the treatment of pharmacoresistant epilepsy, that the MTL additionally plays a specific, causal role in short‐term memory (STM). Patients (n=22) and age‐matched healthy control participants (n=26) performed a STM task with a sensitive continuous report measure. This paradigm allowed us to examine recall memory for object identity, location and object‐location binding, independently on a trial‐by‐trial basis. Our findings point to a specific involvement of MTL in object‐location *binding*, but, crucially, not retention of either object identity or location. Therefore the MTL appears to perform a specific computation: binding disparate features that belong to a memory. These results echo findings from previous studies, which have identified a role for the MTL in relational binding for LTM, and support the proposal that MTL regions perform such a function for *both* STM and LTM, independent of the retention duration. Furthermore, these findings and the methodology employed here may provide a simple, sensitive and clinically valuable means to test memory dysfunuction in MTL disorders.

1

Classical views of the medial temporal lobe (MTL) have established that it plays a crucial role in long‐term memory (LTM; Scoville & Milner, 1957). Here we demonstrate, in a sample of patients who have undergone anterior temporal lobectomy for the treatment of epilepsy, that the MTL additionally plays a specific, causal role in short‐term memory (STM). Patients and healthy control participants performed a STM task with a sensitive continuous report measure. This paradigm allowed us to examine recall memory for object identity, location and object‐location binding, independently on a trial‐by‐trial basis. The results point to a specific involvement of MTL in object‐location *binding*, but, crucially, not retention of either object identity or location. These findings are consistent with results from investigations that have identified a role for the MTL in relational binding for LTM, supporting the proposal that MTL regions perform such a function for *both* STM and LTM (Esfahani‐Bayerl et al., 2016; Olson, Moore, Stark, & Chatterjee, 2006; van Geldorp, Bouman, Hendriks, & Kessels, 2014; Yonelinas, 2013). The methodology used here may provide a simple, sensitive, and clinically valuable means to test memory dysfunction in MTL disorders.

The distinction between short‐ and long‐term memories has been established over many years by studying patients with MTL damage (Baddeley, Allen, & Vargha‐Khadem, 2010; Jeneson, Mauldin, & Squire, 2010; Jeneson & Squire, 2012; Shrager, Levy, Hopkins, & Squire, 2008; Squire, 2017). Contrary to these findings, some neuroimaging and patient studies have presented evidence in favor of a possible role of the MTL in STM (Esfahani‐Bayerl et al., 2016; Olson, Moore, et al., 2006; Olson, Page, Moore, Chatterjee, & Verfaellie, 2006; van Geldorp et al., 2014; Watson, Voss, Warren, Tranel, & Cohen, 2013). In an attempt to reconcile these findings, it has been argued that MTL structures do not play a role in all aspects of STM but perform a specific computation: relational binding of information bringing together disparate elements of an episodic (Davachi, 2006; Eichenbaum, Yonelinas, & Ranganath, 2007) or short‐term memories (Koen, Borders, Petzold, & Yonelinas, 2016; Pertzov et al., 2013).

However, most studies reporting STM deficits in patients with MTL damage have used either set sizes above putative STM capacity limit, long retention durations or did not control for level of difficulty between conditions, leading to proposals that LTM processes might in fact have been involved when performing these STM tasks (Axmacher et al., 2007; Oztekin, Davachi, & McElree, 2010). Here, we aimed to address these concerns by (a) examining memory performance below capacity levels (i.e., 1 or 3 item loads), (b) controlling for encoding of items into memory, and (c) using a sensitive task that provides measures of both feature and binding memory on a trial‐by‐trial basis in a continuous manner rather than using a binary measure. Our findings provide evidence for the role of MTL in STM in a group of patients who had undergone temporal lobectomy for pharmacoresistant temporal lobe epilepsy (details in Table 1 **;** Figure 1a shows lesion overlap). Using a visual STM paradigm that is sensitive to deficits in feature binding, our results provide a more nuanced understanding of STM impairments in patients with circumscribed MTL lesions, which may prove useful to identify and monitor memory impairments in such patients.

**Table 1:** Participant demographics

	Temporal lobectomy	Pathology	Gender (M/F)	Age mean (*SD*)	Years of education mean (*SD*)	Temporal lobectomy (left/right)	Years after surgery mean (*SD*)	ACE mean (*SD*)
*Patients*								
01	Left	TLE, HS	F	48	12	L	8	87
02	Left	TLE, HS	M	41	12	L	8	80
03	Right	TLE, HS	F	46	12	R	1	89
04	Left	MTLE, HS	F	48	12	L	10	91
05	Left	TLE, HS	M	33	14	L	1	82
06	Left	TLE, HS	F	27	16	L	1	97
07	Left	TLE, HS	M	40	14	L	5	94
08	Right	TLE, HS	F	44	11	R	2	95
09	Right	TLE, HS	F	39	12	R	11	82
10	Left	TLE, HS	F	37	16	L	1	82
11	Left	TLE, HS	F	49	16	L	3	87
12	Right	TLE, HS	M	23	13	R	12	75
13	Right	TLE, HS	M	63	17	R	4	97
14	Right	TLE, HS	M	55	14	R	2	92
15	Right	TLE, HS	F	43	12	R	4	91
16	Right	TLE, HS	F	48	17	R	1	97
17	Right	HS	F	47	12	R	19	88
18	Right	TLE, HS	M	38	18	R	4	90
19	Left	TLE, HS	F	21	12	L	2	97
20	Right	Dysembyoplastic Neuroepithelial tumor	F	24	14	R	0	85
21	Left	HS	M	37	14	L	1	81
22	Left	TLE, HS	F	43	18	L	1	97
Overall			8/14	40.6 (10)	14 (2.2)	11/11	4.6 (4.8)	88.9 (6.6)
Controls (*n* = 26)			13/13	36.7 (12.7)	15.6 (3.4)	n/a	n/a	94.4 (6)

HS = hippocampal sclerosis; *SD* = standard deviation; TLE = temporal lobe epilepsy.

**Figure 1:**
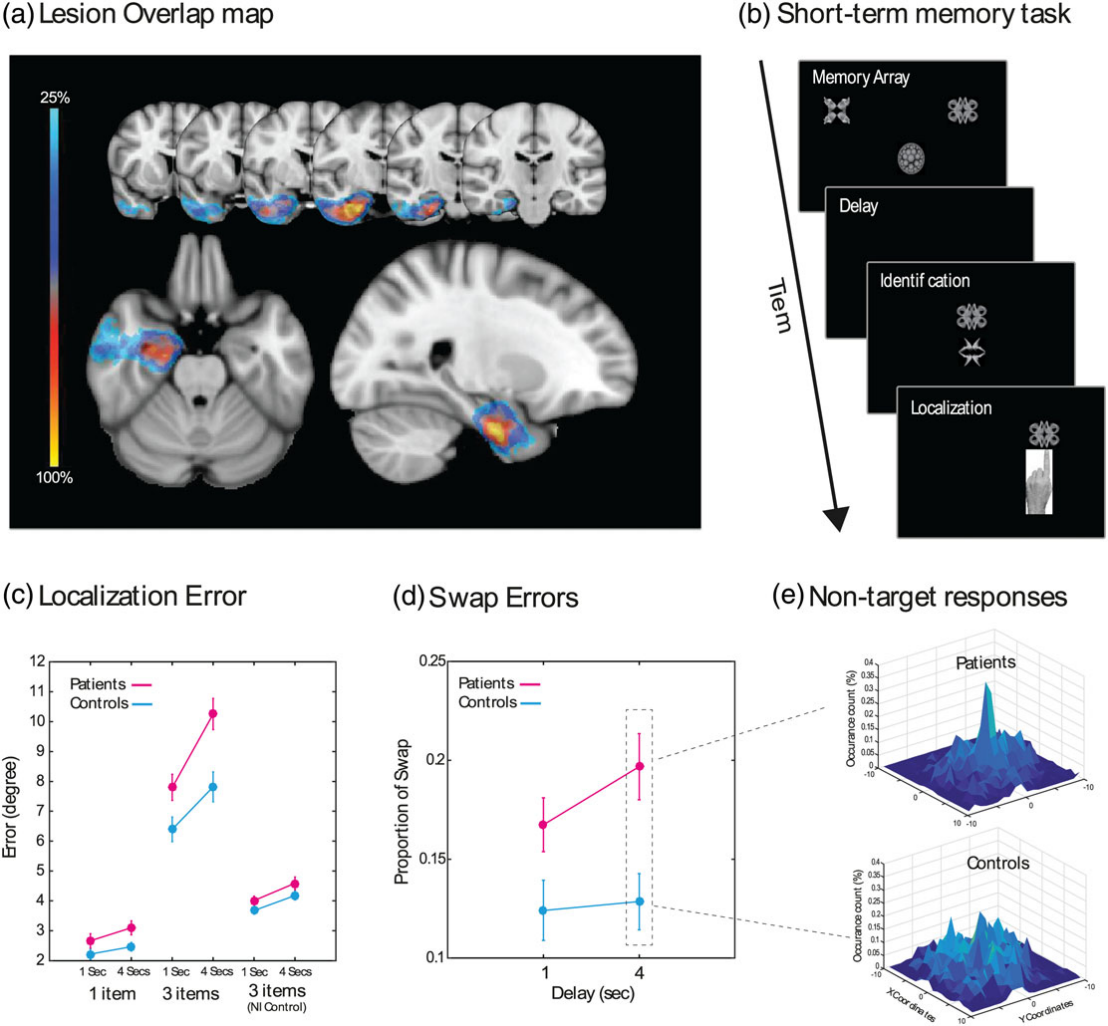
(a) Lesion overlap map: the extent of resection for 20 of the patients is demonstrated here with left lesions flipped onto the right hemisphere, common to at least 25% of all patients. As illustrated, there is high fidelity with regards to the removal of anterior mesial temporal structures. (b) Short‐term memory task: participants were presented with a black and white memory array followed by a delay (1 or 4 s). They were then presented with two fractals, one from the memory array and a foil. On a touchscreen computer, participants first had to touch the fractal they had seen before (in the memory array) and drag it to its remembered location. (c) Localization error: Patients were significantly impaired compared to healthy participants for larger set sizes and in longer delays. Performance between the groups was comparable however after the nearest item control. (d) Proportion of swaps (from total number of trials) in three item conditions, following 1 and 4 s delays. Patients made significantly more swap errors than healthy controls, specifically following 4 s delay. (e) Histogram of nontarget responses in patients and controls following 4 s delays. Centre of the figure corresponds to the location of nontarget (non‐probed) items in memory, thus a response to the non‐probed item in a given trial will translate into a point in the center of the histogram. There is a peak in responses around nontargets in patients but reduced in healthy controls. Error bars represent ± 1 standard error of mean

A schematic of the STM task is presented in Figure 1b. The task was identical to that previously used by Pertzov et al. (2013), except that the fractals were presented in monochrome. In brief, participants were required to keep in mind 1 or 3 fractals and their location on the screen. Fractals in the memory array did not appear at screen center and had a minimum distance of 3.9° of visual angle from the edges of the screen. Following a delay (1 or 4 s), participants were then presented with two fractals, one from the memory array (target) and a foil. They then had to select the fractal previously seen in the memory array (identification accuracy) and drag it to Its location (continuous or analogue measure of localization memory). Participants completed two or three blocks of 50 trials, each lasting ~10–15 min.

The groups (patients vs. age‐matched healthy controls) did not differ significantly in age (*t*[46] = 0.8, *p* = .38) or gender (χ^2^[1, *N* = 48] = 0.9; *p* = .3). Overall patients performed significantly less well on the cognitive screening test, Addenbrooke's cognitive examination or ACE‐III (*t*[46] = 2.99, *p* = .004), and had less years of education (*t*[43.5] = 2.1, *p* = .044) than healthy controls. For all STM analyses, both overall ACE‐III score and years of education were added as covariates. Any differences between patients with right and left lobectomy were examined using side of resection as a between‐subject factor. There was no main effect of side of temporal resection or any interaction between this factor and any of the experimental factors reported below. For further analysis, we have included all patients as one group. For identification and localization memory performance analysis, repeated measures ANCOVA with number of items (1 or 3) and delay (1 or 4 s) as within‐subject factor and participant group (i.e., patients or healthy controls) as a between‐subject factor was used.

Identification performance was significantly worse for larger set sizes and longer delays (main effects *F*[1, 44] = 7.8, *p* = .008, η^2^_p_ = 0.15 and *F*[1, 44] = 4.67, *p* = .036, η^2^_p_ = 0.1, respectively). Importantly however, there was neither main effect of group nor a significant interaction between delay or set size with group (Identification accuracy for set size 1: healthy controls with mean of 98% and standard deviation (*SD*) of 3% and patients with mean of 96% and *SD* of 4%; identification accuracy for set size 3: healthy controls with mean of 90% and *SD* of 7% and patients with mean of 89% and *SD* of 9%). For the remaining analyses, only trials where participants had previously selected the correct item were included.

Localization memory was indexed by the distance between the true and reported location of a fractal. Localization was worse for larger set sizes (main effect of set size: *F*[1, 44] = 24.1, *p* < .001, η^2^_p_ = 0.35) and in patients (main effect of participant group *F*[1, 44] = 5.04, *p* = .030, η^2^_p_ = 0.10). Post hoc t‐tests revealed larger errors in patients for set size 3 after a 4‐s delay (*t*[46] = 3.36, *p* = .002, Figure 1c). This gives rise to a critical question. Is impaired performance simply due to a deficit of memory for location, or is it attributable to identity‐location binding or both?

To address this, we examined maintenance of bound objects in STM, by counting the number of trials in which the fractal was placed within 5° of one of the *other, non‐probed fractal locations*, after controlling for chance probability of obtaining a swap error using the method described by Pertzov et al. (2013). Patients made significantly more swap or misbinding errors compared to healthy controls (*F*[1, 44] = 4.1, *p* = .049, η^2^_p_ = 0.09, Figure 1d).

Follow‐up t‐tests revealed that they made significantly more swap errors following both 1 and 4 s delays (*t*[46] = 2.1, *p* = .042; ns. after correcting for multiple comparisons, using Bonferroni correction and threshold of 0.025 and *t*[46] = 3.1, *p* = .003, respectively). This is also demonstrated in the histogram of responses centered on the nonprobed item locations. On longer trials, there is a peak of responses centered on the location of the nonprobed items in patients but crucially reduced in control participants (Figure 1e).

Can the increase in swap errors observed in 4 s trials *fully* explain impaired localization performance in patients in this condition? To examine this, we calculated localization error with respect to the *closest* fractal that had been in the memory array, rather than the original location of the probed item. That is, we first calculated the difference between the response location and the locations of all items in the memory array. We then chose the smallest error, regardless of whether it was the probed fractal *or* one of the other items in the memory array.

This analysis controls for swap errors, because in trials where a swap occurs, we simply measure the error as the distance between the location to which the item had been dragged and the nearest fractal that had appeared in the memory array. Hence, this is termed the *nearest item control* (NI control; for further details, see Zokaei et al., 2017). After controlling for swap errors using the NI control measure, there was no longer any significant differences between groups on localizations performance (*F*[1, 44] = 3.2, *p* = .08, η^2^_p_ = 0.07, Figure 1c NI control). Therefore, in trials with three items, both patients and healthy participants were making swap errors, as demonstrated by a decrease in localization error following NI control in both groups. Importantly, the difference between the two groups following this analysis disappeared suggesting that the increased localization error in patients was due to increased proportion of swaps, in patients compared to healthy controls.

Together these results highlight a specific impairment in STM associated with MTL lesions. Patients were able to remember object identity (fractals) just as well as controls when examined by a traditional, binary (correct/incorrect) recall measure. However, a deficit emerged when their location memory was assessed using a continuous, analog measure. The lack of a significant increase in swap or misbinding errors with 1 s retention delays demonstrates that impairment in patients cannot be explained by deficits at encoding. Rather, this deficit could be accounted for entirely by an impairment in maintaining object‐location binding. Finally, these deficits were observed at set sizes below putative item capacity limits of STM. Importantly, the deficit emerged when controlling for ACE‐III scores and years of education, thus the differences cannot also be attributed to baseline differences in education or overall cognitive ability between the two groups.

Others have proposed a role for the MTL in relational binding of features belonging to an episode in LTM (Davachi, 2006; Eichenbaum et al., 2007). However, the specific role of MTL implicated here in short‐term binding of object features points to a general role of MTL that extends beyond the classical distinction between cognitive processes of long‐ versus short‐term memories. Indeed, it highlights a computation that might be shared between many cognitive functions, namely, binding of features to perceive and maintain coherent objects. Complementary to this, it has been hypothesized that the MTL plays a crucial role in high‐resolution binding of features for perception as well as STM and LTM (Yonelinas, 2013), for example, for maintenance of complex scenes or tasks that require precise maintenance of recall of bound information (Hartley et al., 2007; Koen et al., 2016). Extending this to the present findings, one might argue that the nature of continuous, analogue tasks (similar to the one used here) inherently requires the maintenance of high‐resolution memory. This becomes specifically apparent when more than one item has to be maintained, resulting in impaired performance in patients with MTL lesions for larger memory set sizes only.

The involvement of the MTL in STM has not always been observed (Baddeley et al., 2010; Eichenbaum et al., 2007; Squire, 2017). Importantly though, in those studies, the tasks used might not have been sensitive to subtle differences between groups, specifically considering the nature of deficit associated with MTL lesions reported here. The design of the current study overcomes any issues of sensitivity by separately measuring recall memory for object identity, memory resolution for locations using a continuous analogue report and the binding between identity and location information. In fact, tasks similar to the one used in this study have successfully been deployed to detect memory deficits in a variety of different patient groups as well as those at risk of developing dementias (Liang et al., 2016; Rolinski et al., 2015; Zokaei et al., 2017).

The present findings are also consistent with results from patients with Alzheimer's disease (AD) and individuals with familial AD due to genetic mutations in *Presenilin 1* or *APP* (amyloid precursor protein). Similar to lesion studies, AD patients and those with familial AD—in whom MTL atrophy has been identified to be a key imaging finding—have difficulty maintaining binding of information even for very short periods of delay (Della Sala, Parra, Fabi, Luzzi, & Abrahams, 2012; Liang et al., 2016; Parra et al., 2009). Moreover, individuals with mutations in the lysosomal enzyme glucocerebrosidase who are also known to have pathological changes to their MTL, demonstrate an increase in swap/misbinding errors in retention of color‐orientation bindings (Zokaei et al., 2014).

In summary, in this study, we demonstrate a causal role of MTL in retention of bound information in visual STM. These findings suggest the MTL is not exclusively involved in LTM but rather supports processes—such as retention of bound features—that are likely to be shared across several cognitive functions. The findings and methodology presented here have important clinical potential. The task provides a quick and easy to administer test of STM that is sensitive to MTL disorders and thereby has the potential to inform clinical practice, by, for example, enabling better detection of subtle memory impairments preoperatively (to enable appropriate counseling of risk) and postoperatively providing targets for interventions to maximize recovery.
